# Pycortex: an interactive surface visualizer for fMRI

**DOI:** 10.3389/fninf.2015.00023

**Published:** 2015-09-29

**Authors:** James S. Gao, Alexander G. Huth, Mark D. Lescroart, Jack L. Gallant

**Affiliations:** ^1^Vision Science Program, University of CaliforniaBerkeley, Berkeley, CA, USA; ^2^Helen Wills Neuroscience Institute, University of CaliforniaBerkeley, Berkeley, CA, USA; ^3^Department of Psychology, University of CaliforniaBerkeley, Berkeley, CA, USA

**Keywords:** fMRI, visualization, python, WebGL, data sharing

## Abstract

Surface visualizations of fMRI provide a comprehensive view of cortical activity. However, surface visualizations are difficult to generate and most common visualization techniques rely on unnecessary interpolation which limits the fidelity of the resulting maps. Furthermore, it is difficult to understand the relationship between flattened cortical surfaces and the underlying 3D anatomy using tools available currently. To address these problems we have developed pycortex, a Python toolbox for interactive surface mapping and visualization. Pycortex exploits the power of modern graphics cards to sample volumetric data on a per-pixel basis, allowing dense and accurate mapping of the voxel grid across the surface. Anatomical and functional information can be projected onto the cortical surface. The surface can be inflated and flattened interactively, aiding interpretation of the correspondence between the anatomical surface and the flattened cortical sheet. The output of pycortex can be viewed using WebGL, a technology compatible with modern web browsers. This allows complex fMRI surface maps to be distributed broadly online without requiring installation of complex software.

## 1. Introduction

**Figure 1:**
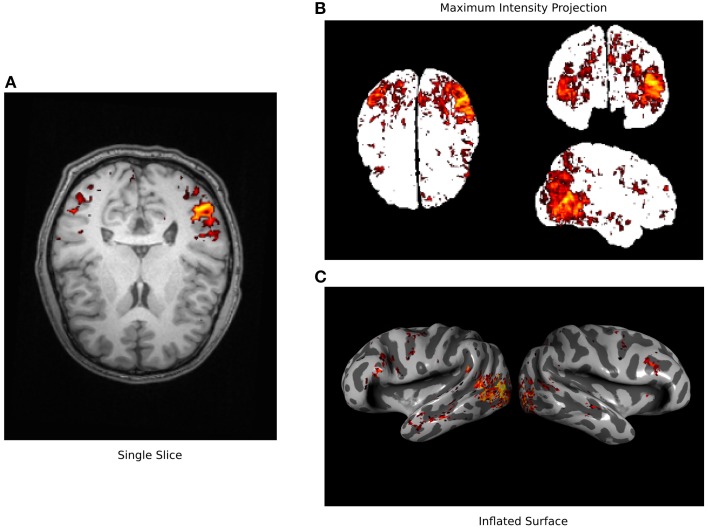
**Typical fMRI visualization methods**. Three typical methods for visualizing fMRI data are used here to visualize a single data set (Huth et al., 2012). **(A)** A single axial slice from an anatomical image is shown overlain with functional data exceeding statistical threshold. It is difficult to recognize anatomical features in this view and much of the functional data is hidden. **(B)** A maximum intensity projection (i.e., a glass brain view) is shown along with all functional data that exceeds a statistical threshold. This view shows more of the functional data than can be seen in the single slice but the anatomical location of these signals is obscured. **(C)** An inflated cortical surface is shown with curvature highlighted in grayscale, and overlain with functional data exceeding statistical threshold. The anatomical location of the functional data is clearer than in the other views, but multiple views are required to see all of the data. None of these standard visualizations show all of the data in a succinct and interpretable way.

Functional magnetic resonance imaging (fMRI) experiments produce rich data revealing the patterns of hemodynamic activity throughout the brain (Huettel et al., 2009). However, tools for visualization of fMRI data remain relatively primitive. Volumetric views that show single slices or maximum intensity projections (Figure 1) reveal only a small portion of the available data. More sophisticated tools use 3D reconstructions of the cortical surface to create inflated or flattened cortical surfaces (Cox, 1996; Goebel, 1997; Dale et al., 1999; Van Essen et al., 2001). However, most of these tools produce static views of the data so it is often difficult to interpret the relationship between cortical anatomy and inflated and flattened surfaces. Furthermore, current packages use standard computer graphics libraries that are not optimized for accurate visualization of volume projections. They tend to under-sample the underlying volumetric data and do not produce optimal visualizations. Finally, no current visualization packages provide a convenient platform for creating interactive online visualizations for a broad audience.

We addressed the shortcomings of 3D visualization tools by developing pycortex, an interactive software package for viewing fMRI data that is optimized for displaying data on the cortical surface. Pycortex streamlines the process of surface visualization and produces interactive displays that switch smoothly between folded, inflated, and flattened views of the cortical surface. Pycortex implements a pixel-wise mapping algorithm for projecting volumetric data onto the cortex. This method samples the underlying functional data densely and produces accurate, visually appealing renderings of the fMRI data. Finally, pycortex uses WebGL to display the results of the analysis. These WebGL-based visualizations can be created and viewed on the fly, or they can be saved as a web page that can be viewed by anyone with a modern web browser. These standalone visualizations can easily be shared with colleagues, included as links in published articles, or shared online with a broad audience.

To see a demonstration of what can be achieved with pycortex, please visit http://www.gallantlab.org/pycortex/retinotopy_demo. This demo shows the results of a retinotopic mapping fMRI experiment that was performed on one subject. Retinotopic mapping is one of the workhorse tools in fMRI experiments of human vision, and it is used to identify the cortical extent and distribution of many human visual areas (Sereno et al., 1995; Engel et al., 1997; Sereno, 1998). In this online demo, the retinotopic mapping stimulus that the subject saw appears on the right, and measured blood oxygenation level-dependent (BOLD) responses measured across the cortical sheet are shown at left.

The rest of this paper is divided into three sections. The first section describes the advantages of cortical surface-based analysis and visualization over other methods. The second explains the innovative aspects of pycortex as a tool for surface visualization. Finally, the third section describes a typical pycortex workflow and presents examples of the major features in pycortex. Readers are encouraged to download the package (https://github.com/gallantlab/pycortex/) to follow along. Additional in-depth examples and explanations are included in the pycortex documentation (http://gallantlab.org/pycortex/docs/).

## 2. Background

fMRI generates rich volumetric data which can be difficult to visualize. Imaging data are often presented as 3D projections onto 2D planes. However, contiguous functional domains in volume visualizations may appear as unconnected patches. Surface visualization provides an intuitive way to simultaneously view all cortical activity recorded in an fMRI dataset (Van Essen et al., 2001). The organization of the mammalian cortex ensures that discrete functional domains can be visualized as contiguous patches on the cortical surface (Felleman and Van Essen, 1991; Kaas, 2012). However, the folding of the cortex obscures information deep in sulci, so functional information is difficult to visualize on the raw surface. To permit better visualization, surface visualizations commonly unfold the sulci and gyri while maintaining anatomical contiguity.

Many fMRI data analysis packages include a surface visualization module, and these all make use of a standard three-step pipeline: (1) a triangular mesh representation of the cortical surface is extracted from an anatomical scan; (2) functional and anatomical data are coregistered; (3) functional data (or the results of some analysis of the functional data) are projected onto the cortical surface mesh representation. In the following sub-sections, we detail how each of these steps is accomplished.

### 2.1. Cortical surface mesh generation

The cortical surface is usually modeled as a triangular mesh in 3D. The mesh is created by first segmenting the brain at the tissue boundaries in a volumetric anatomical scan, then applying a mesh generation algorithm such as marching cubes (Dale et al., 1999). Once the triangular mesh has been created, 3D geometrical operations are performed to inflate and flatten the cortical surface (Fischl et al., 2001). Flattened views of the cortical surface show data across the entire cortex without the need for multiple views in 3D. In order to create a flattened cortical surface representation from the three-dimensional cortical sheet without introducing excessive spatial distortion, relaxation cuts must be introduced into the cortical surface model. This operation is typically performed manually. To avoid splitting regions of interest on the flattened surface it is best to use functional localizer information when determining the location of relaxation cuts.

### 2.2. Coregistration

Functional MRI data are typically collected using an imaging sequence that is optimized for functional rather than anatomical tissue contrast (Nishimura, 2010). Thus, the functional data and the anatomical data that produced the surface must be spatially aligned before projecting the functional data onto the cortical surface model. This process is called coregistration (Jenkinson and Smith, 2001) and results in a transformation matrix that maps between the 3D coordinates of voxels in the functional data and the 3D coordinates of voxels in the anatomical data. Coregistration is typically performed automatically by global optimization of an affine transform from the functional image to the anatomical image used to generate the surface (Jenkinson et al., 2002). Since the contrast between anatomical and functional images are different, these frequently generate poor alignments.

In contrast, recent coregistration algorithms optimize surface intersections with the functional data. By maximizing the gradients across the surface, these algorithms can achieve accurate coregistration with no manual intervention. This technique—called boundary based registration (BBR) (Greve and Fischl, 2009)—performs extremely well for data collected using a whole-head slice prescription. However, BBR can still fail unexpectedly. Imaging artifacts related to echo-planar imaging such as distortions and dropout negatively affect the performance of BBR, and it rarely works well with partial-head slice prescriptions. This is why the accuracy of automatic coregistration should always be verified visually by overlaying the transformed functional image on the anatomical data.

In most fMRI analysis pipelines, the functional-anatomical transformation estimated by the coregistration procedure is used to re-slice functional data into the same space and resolution as the anatomical scan. Re-slicing allows interpretation of functional results with respect to volumetric anatomical landmarks and provides a straightforward means of transforming data into standardized anatomical spaces (e.g., MNI or Talairach space) (Friston et al., 1995). Re-sliced data can also be projected onto the inflated or flattened cortical surface. However, as we will describe in more detail below, re-slicing data into volumetric anatomical space is not strictly necessary for projection of the data onto the cortical surface. Only the functional-anatomical transformation is necessary.

### 2.3. Projection of functional data

**Figure 2:**
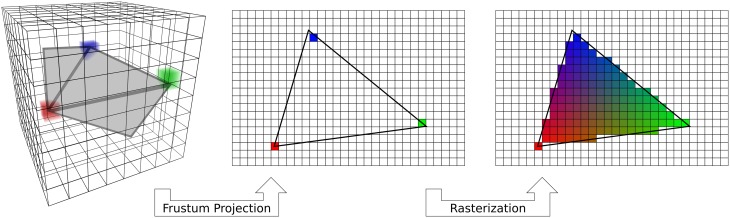
**Standard vertex-based mapping**. Standard OpenGL rendering implements an algorithm that can be used for vertex-based mapping. First, a fragment of the surface (gray) embedded in the voxel data (3D grid) is used to sample the data. Sampling occurs at only the vertices of the triangle (red, green, blue cubes). The surface triangle is projected to the screen on a regular 2D grid through a standard frustum projection (Woo et al., 1999). Only the data sampled by the vertices are carried over to the screen. Finally, the automatic graphics pipeline rasterizes the triangle by interpolating the colors across a barycentric coordinate space. Vertex-based mapping is generic, but does not adequately sample volumetric data.

Visualization of the functional data on the cortical surface is usually accomplished using a 3D graphics pipeline that implements simple vertex-based projection. Vertex-based projection (Woo et al., 1999) can be split into three steps (Figure 2). First, each vertex in the cortical surface mesh is mapped into the functional volume. Second, the volumetric functional data are sampled at the vertex locations. If functional data have been re-sliced to anatomical space then this is trivial. However, by using the functional-anatomical transformation information this mapping can be applied directly from the functional data (in its native space) onto the cortex without re-slicing. Finally the color of each pixel on the display is determined by a 3D renderer, usually by linear interpolation between the values of the nearest vertices. This three-step method is not optimal because it requires two separate sampling steps: once from volume space to vertex space, and then again from vertex space to display (pixel) space. If the data are re-sliced to anatomical space then this adds a third sampling step. Each sampling step leads to aliasing and loss of resolution. Furthermore, mesh smoothing and other surface manipulations may cause uneven vertex spacing, and therefore uneven spatial resolution across the cortical surface. This effect is especially apparent with high resolution data (for example, < 2mm^3^ voxel data collected using 7T fMRI).

One possible way to address this problem is to subdivide the mesh surface to increase the number of vertices, and thus the number of sampling locations. This is the solution is adopted by BrainVoyager QX (Goebel, 1997). However, increasing the number of vertices greatly increases the computational load, and can only be applied to small portions of the cortex at one time.

## 3. Innovations in pycortex

Pycortex improves the process of fMRI visualization in a number of ways. First, pycortex integrates a number of tools to generate high quality cortical surface reconstructions. Pycortex uses these surfaces to sample functional data using a novel projection algorithm that results in much higher resolution visualizations. Finally, we draw on the power of modern graphics cards to provide a highly interactive, accurate, and portable visualization platform that works from within any modern web browser.

### 3.1. Surface generation and coregistration

Software packages such as Caret (Van Essen et al., 2001), Freesurfer (Dale et al., 1999), SUMA (Cox, 1996), and BrainVoyager QX (Goebel, 1997) are typically used to generate a high quality mesh representation of the cortex. Since these surface segmentation and mesh manipulation algorithms are already well developed, they are not reimplemented in pycortex. Instead, pycortex uses surface information output from these packages to create three-dimensional visualizations that can be easily manipulated and viewed. Pycortex is most closely integrated with Freesurfer, a free, open-source software package that is already used by a large community [including the Human Connectome Project (Glasser et al., 2013)]. However, pycortex can import most of the 3D formats that are used by standard MRI segmentation packages (see documentation for details).

Since the advent of BBR, automatic coregistration algorithms usually produce high quality alignment for whole-brain studies without manual intervention. However, when partial-head slice prescriptions are used then it is best to perform manual coregistration, and it is always wise to visually check any coregistration solution. Pycortex provides an alignment tool that plots the surface mesh overlaid on the functional data. This allows users to view the alignment in orthogonal slice planes (to simulate traditional piecewise linear transformations), or using a global 3D view. The surface may be translated, rotated, and scaled interactively relative to the functional volume. The user can use these tools to visually match the surface with the underlying functional volume.

Some of the available tools for coregistration and segmentation are difficult to use. For example, the Freesurfer interface for marking relaxation cuts for surface flattening can be cumbersome; the 3D interface uses button controls for rotation and manipulation rather than a much more natural click and drag interface. Therefore, pycortex integrates several different tools to simplify the process of segmentation and coregistration. Pycortex replaces the default Freesurfer tool with Blender, an open source mesh editing program that is relatively easy to use. Pycortex also integrates with the BBR implementation provided by FSL to provide automatic coregistration that is compatible with surfaces generated by Freesurfer. The simple command pipeline provided by pycortex makes the entire process of surface generation and visualization smooth and relatively straightforward.

### 3.2. Pixel-based mapping

**Figure 3:**
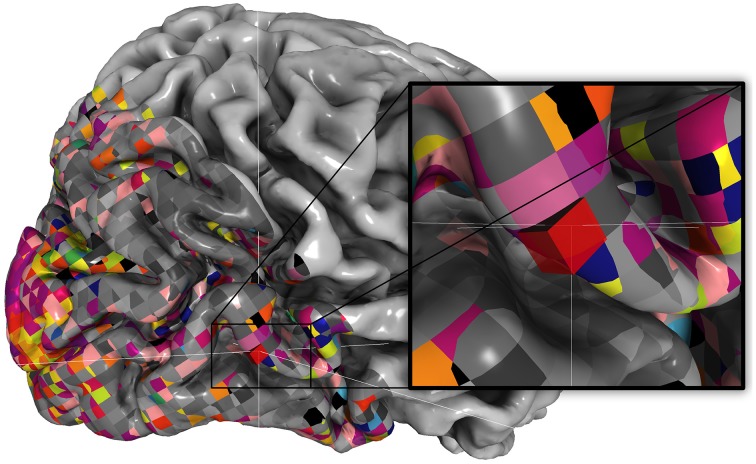
**Retinotopic data for example subject**. Pixel-based mapping in pycortex renders voxels true-to-form. Here, a retinotopic map as in Hansen et al. (2007) is plotted using webgl. Note that the slanted slice prescription and the isotropic voxel size is easily visible due to pixel-based mapping and nearest-neighbor sampling. The inset shows how a single voxel intersects the surface.

**Figure 4:**
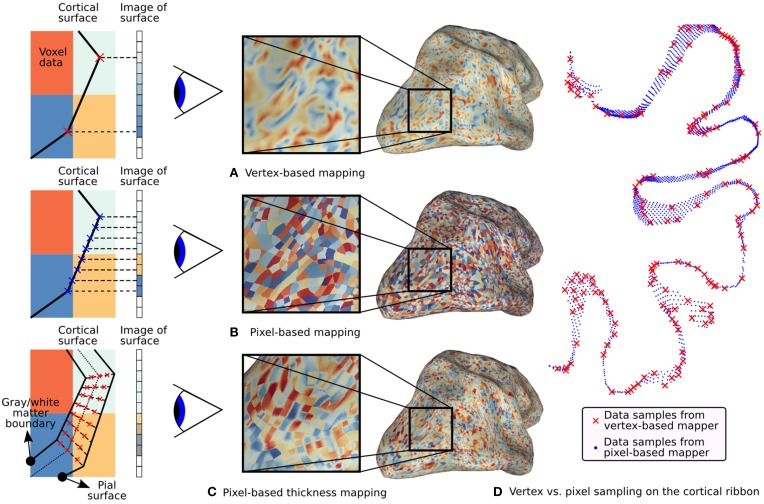
**Typical fMRI data projections**. On the left, simulated 2D volumes are projected onto a 1D screen. The large colored blocks represent voxel data and the small rectangles represent screen pixels. Three different mapping methods are illustrated along with their effect on the surface. **(A)** Standard vertex-based mapping uses vertices in the surface mesh to sample the underlying functional data. The vertices (red x's) sample the functional data using a nearest-neighbor algorithm and the values are automatically interpolated by the rasterizer. Although the surface passes through the orange voxel at bottom right, there are no orange pixels on the screen because there is no enclosed vertex. **(B)** Pixel-based mapping projects screen pixels into the volume to sample the underlying data. Orange pixels now appear on the screen since the surface passes through that voxel. **(C)** Thickness mapping samples data along the entire line between the white matter and the pial surface, thereby reflecting activity throughout the thickness of the cortical mantle. **(D)** The difference in sampling density between pixel-based and vertex-based mapping is shown for a 1mm slice through the cortical volume as it is mapped onto a flat map. Vertex samples are shown as red x's and pixel samples are shown as blue dots. The increased density improves the accuracy of functional data display, particularly with high-resolution functional data.

As discussed above, vertex-based mapping can be a lossy process that involves unnecessary interpolation. Pycortex implements a simpler, more accurate sampling scheme called pixel-based mapping (Figure 3). This scheme replaces the three separate projection steps with a two-step process that only samples the data once. This pixel-based algorithm directly maps the pixel coordinates on the display into the functional volume, thereby eliminating the intermediate vertex space representation. Pixel-based mapping therefore produces much higher fidelity images of the underlying data than those produced by the typical vertex-based method (Figure 4A vs. Figure 4B). Compared to vertex-based methods, pixel-based mapping samples the cortex much more densely (Figure 4D). However, it is computationally costly since the functional volume must be resampled for every viewpoint in 3D. If the view is rotated even a degree, every pixel must be mapped anew into volume space. However, pycortex renders visualizations smoothly and in real time when used with modern graphics cards and shader pipelines.

Once pixel locations are mapped into volume space, they must sample the underlying data to derive their color. Different sampling methods trade off between speed and accuracy and generate visually distinct images. Pycortex includes several different sampling methods which allow very fine-grained control over this trade-off. The simplest method is nearest-neighbor sampling in which the mapped pixel is assigned the value from the nearest voxel. Nearest-neighbor sampling is fast and easy to compute and simple to interpret. However, nearest-neighbor sampling renders hard edges between adjacent voxels, so it can create a false impression of sharpness in the data. Trilinear sampling interpolates between the eight closest voxels to compute each sample. Trilinear interpolation uses a triangular filter that reduces aliasing compared to nearest-neighbor sampling. However, this suppresses high spatial frequency information and may produce results that are too smooth. Sinc filtering results in samples with the lowest reconstruction error (Oppenheim and Willsky, 1996). Sinc filtering can be approximated with a lanczos filter that optimally preserves the spatial frequencies present in the functional data. However, this truncated filter is slow to apply so this sampling scheme cannot be used for real-time rendering. Other sampling schemes can also be implemented in pycortex through an extensible interface.

Although pixel-based mapping shares some superficial similarities with ray tracing, its operation is simpler and faster than a full ray tracing pipeline. In ray trace rendering, simulated rays are projected from a virtual camera through screen pixels. If the ray intersects with a mesh within the scene, the pixel takes on the color of the underlying geometry given the lighting model in use. Pixel-based mapping also computes the color on a per pixel basis, however it is strictly a fragment shading procedure (Woo et al., 1999). Geometric operations are performed by frustum projection, rather than ray intersection.

With other visualization packages such as Caret, only a single position between the pial and white matter surface (typically halfway between) is sampled to generate the visualization. However, human cortex varies in thickness from 1.5 to 3 mm (Fischl and Dale, 2000); thus sampling only a single position may ignore voxels which are closer to the white matter or pial surface. Pycortex uses a special sampling scheme called thickness sampling to take multiple samples between pial and white matter surfaces, which captures activity distributed through the thickness of the cortex. In thickness sampling, each pixel is mapped to a line in volume space that stretches between the pial and white matter surfaces. Several samples are taken along this line and the samples are averaged to derive the final pixel value (see Figure 4C). Alternatively, a single plane within the cortical mantle can be selected so that the data can be viewed anywhere between the pial and the white matter surfaces. Thickness sampling is a costly process to run on a CPU, but it is fast and efficient when implemented using custom shaders and a modern graphics card. To further improve responsiveness with thickness sampling, samples along a random set of positions through the cortical sheet can be averaged. This dithering trades off accuracy in favor of interactivity, but still accurately represents information through the thickness of the cortex.

### 3.3. WebGL and data sharing

Graphics card acceleration allows highly complex datasets to be rendered in real time on standard computers. Typical 3D data visualization software relies on programming interfaces like OpenGL to access this powerful hardware. However, software which relies on OpenGL typically requires extensive installation procedures to visualize even simple datasets. WebGL is a new technology which melds the OpenGL programming interface with Javascript, a language used to program websites. This allows powerful data visualizations to be programmed directly inside a web browser. Bringing graphics card acceleration to web pages provides the opportunity to create portable, interactive visualizations of fMRI data.

Pycortex takes full advantage of the power of WebGL by implementing custom shaders on the graphics card. Modern graphics cards include programmable shaders that allow custom code to be uploaded to the card, thus enabling highly parallel rendering operations. By using custom shaders, pycortex can use accelerated rendering algorithms that would otherwise be too slow to be practical. When data is visualized in the WebGL view, only volumetric data and surface structure is passed into the web browser; all other functionality is accomplished by shader programs. Custom shaders included in pycortex enable the surface to be drawn quickly, even when pixel-based mapping and a user-selectable sampling method are used.

Because web browsers are ubiquitous on modern personal computers, no special installation is required to view pycortex visualizations. The use of a web browser as the front end for pycortex also allows an unprecedented level of interactivity. For example, the anatomical surface can be flattened interactively simply by dragging a slider. This interactive design helps the user to develop a clear sense of the correspondence between flattened and folded surfaces. Pycortex can also display temporally varying time-series data on the cortical surface in real time. This allows simultaneous visualization of the experimental paradigm and the functional data in real time (for an example of such a visualization, see http://www.gallantlab.org/brainviewer/retinotopy_demo).

It is simple to post pycortex visualizations to a web page for public viewing. These static visualizations are generated using a simple command that generates a single web page with most resources embedded directly. The surface structure, data, and the webpage can then be posted to any public facing web site. For example, the online Neurovault data repository (http://neurovault.org) now makes use of pycortex, and any fMRI data uploaded to Neurovault can be visualized automatically in pycortex. These visualizations are visible at a static web address that can be referenced in papers and shared with anyone with a web browser.

## 4. Pycortex functionality

Pycortex is free, open-source software written in python and javascript. Pycortex adds to the growing body of python tools for neuroscience (Millman and Brett, 2007; Halchenko and Hanke, 2012; Pedregosa et al., 2012). Additional third-party software used by pycortex to provide optional and core functionality is outlined in Table 1. Installation instructions for pycortex and associated software can be found at http://pycortex.org.

**Table 1:** **The following software is used in pycortex for either core or added functionality**.

**Software**	**Function**
Python	Required to run pycortex
ImageMagick	Flatmap ROI rendering
Freesurfer	Surface segmentation and generation
Blender	Surface manipulation for flattening
Inkscape	ROI definition and manipulation

*Please consult the pycortex documentation for additional information about the installation process*.

In pycortex user interaction is handled through the python command line. Here, we present the typical workflow for pycortex, proceeding from anatomical and functional images to a web-based 3D visualization. In the simplest possible case, only three commands are required to generate a fully interactive surface visualization in pycortex:


>>> cortex . segment . init_subject( " S1 " , 
                        " T1_anatomical . nii.gz " )
>>> cortex . align.automatic( " S1 " , 
      " transform_name " , " functional . nii.gz " )
>>> cortex . webshow (( data, " S1 " , 
                               " transform_name " ))


These commands illustrate three important python modules for cortical segmentation and visualization. The segment module initializes the cortical segmentation using an anatomical image. The align module provides both automatic and manual coregistration tools for coregistering the surface and functional images. The webgl module is used to generate interactive web visualizations. Two other modules are also documented here to highlight additional pycortex functionality; the overlay module can be used to define surface overlays and regions of interest (ROIs), and the quickflat module is used to generate figure-quality images.

**Figure 5:**
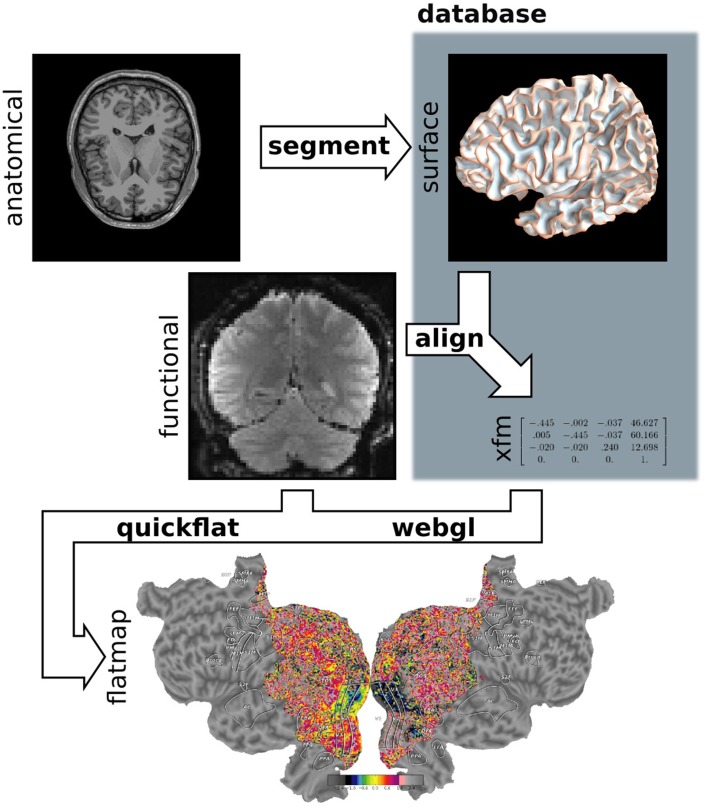
**Pycortex module diagram**. Pycortex provides an integrated visualization toolkit for fMRI. Several pycortex modules are used to transform the user-provided anatomical and function data into an interactive visualization. The segment module integrates with Freesurfer to generate the surface. The align module uses the functional data and the surfaces to generate a transform. The quickflat and webgl modules generate static 2D flatmaps and interactive 3D visualizations, respectively. The overlays module is used to generate vertex-based ROIs and extract surface-defined volume ROIs.

Pycortex makes use of a large amount of internal data such as subject surfaces, alignments and other metadata. All data required for pycortex is kept in a database that is implemented as a simple directory on the user's hard drive. In most cases pycortex uses this database seamlessly without requiring any interaction from the user. (For more information about the database and all supported file formats, consult the pycortex documentation.) A summary of the data flow in pycortex can be found in Figure 5.

### 4.1. The segment module

Pycortex integrates with Freesurfer to generate surfaces from anatomical images. For optimal results from Freesurfer, anatomical MRI images should be collected using the scanning protocols defined in the Freesurfer documentation ^1^. Freesurfer is optimized to work with a specific multi-echo T1 scan sequence, and we have found that this sequence maximizes surface quality and minimizes the need for manual editing of the surface after automatic segmentation. Once the requisite anatomical images have been collected, only two lines of code are necessary to initiate surface segmentation in pycortex:

>>> **from** cortex **import** segment
>>> segment . init_subject( " S1 " , 
                   " T1_anatomical . nii.gz " )

This command uses Freesurfer to generate the surface files automatically and stores the surface in the pycortex database with the subject identifier "S1". (All further pycortex processing steps will refer to this surface by the subject identifier assigned at this stage of processing). Segmentation is a slow process that can take up to 12 h for one subject on a Intel Core i7 2700k, but it can be run unattended.

Freesurfer generally performs very well on normal brains, but minor topological errors may occur in areas of low contrast, such as at the cerebellar boundary and around the optic nerve. Segmentations may also be compromised around lesions that may be present in diseases such as stroke or aneurysm. It is therefore wise to check all surfaces before further processing using one of the following commands:

>>> segment . fix_wm ( " S1 " )
>>> segment . fix_pia ( " S1 " )


These commands open an interface that permits segmentation edits to be applied directly to the white matter or pial surfaces. One window is from Freesurfer's segmentation editor tool; white matter voxels can be added or removed in this interface to alter the final surface. A 3D view also opens in another window to view the surface that resulted from the current segmentation. Minor segmentation errors typically manifest as spikes or lumpy areas on the surface. Having both interfaces open simultaneously allows location information to be shared, facilitating manual editing to improve surface extraction. (For more information about how to make these edits, consult the Freesurfer documentation, or follow the segmentation tutorial in the pycortex documentation.) Saving and exiting from all windows will automatically run Freesurfer once more to apply changes and generate new surfaces.

Once the surfaces are deemed satisfactory, relaxation cuts can be introduced to facilitate creation of cortical flatmaps. This is accomplished with one command:

>>> segment . cut_surface( " S1 " , " lh " )

This command automatically exports the surface and opens it in Blender. Vertex selection and face deletion tools can be used to remove the medial wall. Vertices can be marked in conjunction with functional data to facilitate relaxation cutting. For example, retinotopic mapping data can be projected onto the brain to facilitate cutting along the calcarine sulcus to separate visual hemifields. Marked cuts are processed automatically for use in the flattening procedure. (In-depth instructions on performing this step can be found in the documentation for pycortex.) When the changes are saved, pycortex automatically flattens the surface and makes the new flat surface available for visualization. Functional data can immediately be plotted on this flatmap.

If segmentation is performed outside of the segment module, it is still possible to use these surfaces in pycortex. For example, if the user has existing surfaces generated by CARET, copying the surface files directly into the pycortex database allows them to be used in any pycortex visualization. (For more information about how to use external surfaces, please consult the pycortex documentation.)

### 4.2. The align module

To project functional data onto anatomical surfaces accurately the functional data must first be coregistered with the anatomical surface. Pycortex supports automatic coregistration using the BBR tool within FSL (see Background). Pycortex also provides a fully manual alignment tool. Three arguments are required to launch the automatic coregistration tool: the subject identifier, the name of the transform, and a functional reference image. For example,

>>> **from** cortex **import** align
>>> align . automatic ( " S1 " , " test_alignment " , 
                       " reference_epi . nii.gz " )

This will automatically coregister the function image with the surface, and store the transform into the pycortex database. After an automatic coregistration, the transform can (and should) be checked with the manual alignment tool to ensure accurate coregistration:

>>> align . manual ( " S1 " , " test_alignment " )

This manual alignment tool has three panels that show the current surface slice intersection with the reference image, and a fourth panel that shows the full 3D rendering with slice positions. Showing the data this way facilitates accurate alignment of the gray matter with the functional data. A sidebar contains options to adjust the contrast and brightness, along with some additional settings. The surface can be moved using key commands listed in the sidebar. Hotkeys and buttons in the graphical interface allow the anatomical volume to be translated, rotated and scaled in order to align it optimally with the functional data. The alignment can be saved using a button or by exiting the interface.

Transforms in pycortex are stored in the pycortex database in the form of an affine transform matrix that operates in magnet isocenter right anterior superior (RAS) space (as defined by NIFTI headers). The matrix transforms surface coordinates, which are typically stored with respect to the anatomical space, into the functional space. This format is compatible with AFNI's transform format. Utility functions are included to allow conversion between AFNI/pycortex format and the FSL format.

### 4.3. The quickflat and webgl modules

**Figure 6:**
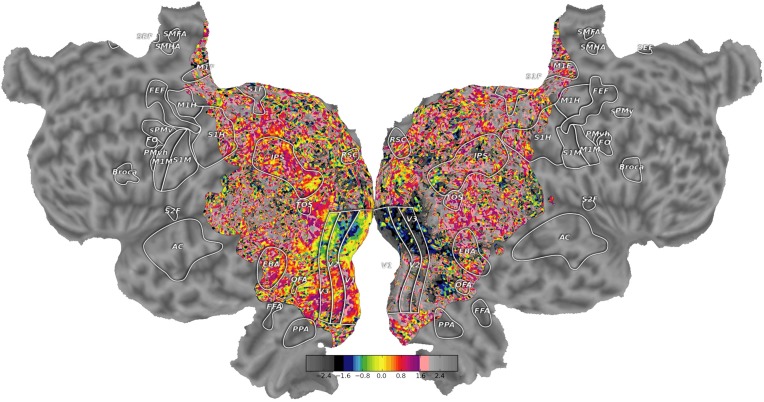
**High quality flatmaps generated by pycortex**. Cortical flatmaps can be quickly generated in pycortex using the quickflat module. Matplotlib is used to view figure-quality flatmaps in a standard size. Options are available to include a colorbar, ROI outlines, ROI labels, and curvature information. This figure shows retinotopic organization of visual cortex for one subject as in Hansen et al. (2007)

Pycortex provides two visualization tools to plot functional data on surfaces. quickflat visualizations use matplotlib to generate figure-quality 2D flatmaps and webgl uses a web browser for interactive visualizations. Both tools use pixel-based mapping to project functional data onto the cortical surfaces accurately.

quickflat was used to generate the figures in Figure 6; to load the same visualization,

>>> **import** cortex
>>> dataset = cortex . load 
                        ( " S1_retinotopy . hdf " )
>>> cortex . quickshow (dataset . angle)

This sequence of commands loads the example dataset (http://gallantlab.org/pycortex/S1_retinotopy.hdf) and then plots the flatmap in a matplotlib window. (For more information about additional display options including options to select the sampling function, please consult the pycortex documentation.)

To generate these flatmaps quickly, the quickflat module precomputes a mapping from volumetric samples to figure pixels. These mappings are represented as sparse matrices so visualizations can be generated from new data quickly by taking the dot product of the matrix with the unraveled volume. To generate these sparse matrix mappings, a grid of pixel locations are generated that span the extent of the flatmap surfaces. A Delaunay triangulation is then generated for the flat surfaces and the simplex membership is found for each pixel. Next, the barycentric coordinate on the simplex is generated from the triangulation transform for every pixel. The original surface coordinate is then computed by substituting the mid-cortical vertex (or averaged across multiple depths for thickness sampling) for the flatmap vertex in the Delaunay triangulation, and weighting the vertices with the barycentric coordinate. Finally, a sampler argument determines which function is used to sample the 3D coordinate.

The webgl visualization can be launched using syntax similar to that used for the quickflat visualization:

>>> **import** cortex
>>> dataset = cortex . load ( " retinotopy . hdf " )
>>> cortex . webshow(dataset)

This starts a web server in python and opens a browser window to display the visualization. After a brief loading period, the cortical surface is shown with the retinotopy demo data projected on the surface. The rendered 3D view is a fully dynamic visualization that allows real time rotation, panning, and scaling.

The data display can be modified interactively in numerous ways. The dynamic view has two sliding windows that contain display options. The large slider at the bottom linearly interpolates the shape of the cortical mesh between the original (folded) anatomical, inflated, and flattened surfaces. This allows the unfolding process to be visualized continuously, and it clarifies the correspondence between 3D anatomical features and the cortical flatmap. The sliding window located at the top contains options that change how the data is displayed. Different colormaps can be selected and the colormap ranges can be altered dynamically. 2D colormaps are also supported, allowing two datasets to be contrasted simultaneously. Multiple datasets can be loaded and compared directly by simply toggling between them. Sliders are provided to change the transparency of the dropout, overlay, data, and curvature layers.

As explained earlier, pycortex uses custom shaders that implement pixel-based mapping. During 3D graphics rendering, the color of each pixel is determined by some predefined code at the fragment shading step. Under a traditional fixed-function pipeline, fragment shading is performed by a rasterizer that implements vertex-based mapping (Woo et al., 1999). In contrast, the fragment shader in pycortex projects each pixel into the functional space in 3D, and then samples the underlying volume data by reading from a texture. Nearest-neighbor or trilinear sampling is automatically performed by OpenGL when the data is read from the texture. This generates a fully interactive and accurate real-time visualization.

The webgl module contains code that parses and generates the HTML and javascript code required to display surface data in a web browser. It provides two possible use cases: a dynamic view that can be controlled by a back end python web server, and a static view that generates static HTML files for upload into an existing web server. The OpenCTM library (Geelnard, 2009) is used to compress the surface mesh into a form that can be utilized by the web browser. If a dynamic view is requested, the webgl module sets up a local web server with all the required surface and data files accessible to the web browser. If a static view is requested, all HTML and javascript code is embedded into a single HTML document and saved to a set of files. Data (in the form of compressed mosaic images) and surface structures are stored separately. These standalone visualizations can then be copied to a web server to be shared with colleagues, included as links in published articles, or shared online with a broad audience.

**Figure 7:**
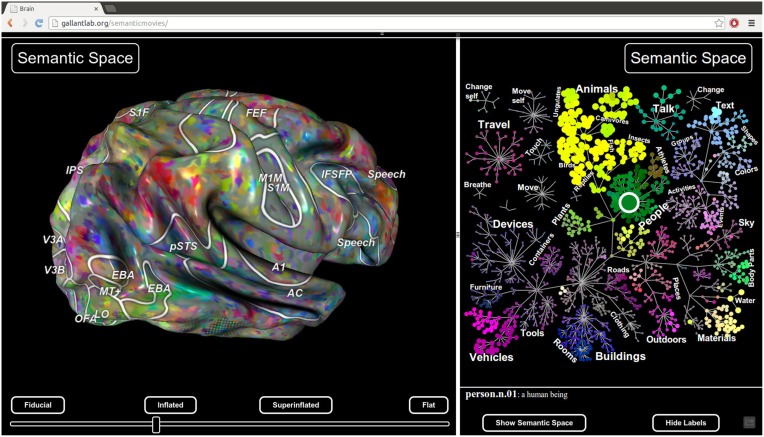
**Static view for web presentation**. Pycortex uses WebGL to generate a static view that can be hosted on a web site. The static view allows users to share data with colleagues, collaborators, and the public. No additional software needs to be installed. This figure, published with Huth et al. (2012), shows a typical static view. A static plugin written in javascript on the right interacts with the 3D view, allowing dynamic interaction between the plugin and the view.

Pycortex also includes a javascript plugin architecture that allows new interactive visualizations to be developed easily. For example, the static viewer released with Huth et al. (2012) http://gallantlab.org/brainviewer/huthetal2012/ contains a plugin that allows the user to visualize how 1765 distinct semantic features are mapped across the cortical surface (Figure 7). Clicking a point on the brain picks the closest voxel and the viewer displays the semantic category tuning for the associated voxel.

Finally, pycortex provides a bi-directional communication framework between python and javascript, so that actions in javascript can be scripted and manipulated in python. This powerful interaction dynamic allows exploratory data analysis in a way never before possible for fMRI.

### 4.4. The overlays module

One common requirement of fMRI studies is to visualize regions of interest (ROIs). ROIs are typically defined in volume space, using a statistical threshold applied to a functional localizer contrast (Poldrack, 2007). Because these thresholded regions are not anatomically constrained their intersection with the cortical surface is not guaranteed to be contiguous or smooth. Another common requirement is to visualize retinotopic ROI defined by identifying hemifield inversions on a cortical flat map (Hansen et al., 2007). The overlays module provides a means to define overlays, such as ROI borders and other surface markers, directly on the cortical surface. These ROIs are automatically rendered by pycortex as paths or regions on the rendered surfaces.

To add an ROI, the user must provide contrast data and a named transform:

>>> **import** cortex 
>>> cortex . add_roi ( ( contrast_data, 
     " S1 " , " fullhead " ) , name = ‘ROI name’, no $ )

This automatically starts Inkscape, an open source vector editing program. A flatmap as generated by quickflat is shown with multiple layers corresponding to different overlays. If a closed path is drawn into the ROI layer, pycortex regards it as a complete ROI. A simple utility function can then extract the volumetric mask of this ROI:

>>> mask = cortex . get_roi_mask, no $ ( " S1 " , 
                        " fullhead " , " V1 " )
>>> mask[‘V1’].shape
(31, 100, 100)

This returns a volume that indicates the number of ROI vertices within each voxel. The volume can be converted into a binary mask by finding all nonzero voxels. This simple thresholding is equivalent to a nearest-neighbor sampling. Pycortex also provides other projection options that may include additional voxels. (For more information, please consult the pycortex documentation.)

Pycortex stores overlays as 2D vector paths in the standard SVG image format that is easily parsed by many libraries. This allows flexible handling of surface overlays either in pycortex (via Inkscape) or in other programs outside of pycortex. Each functional contrast added with cortex . add_roi is stored as an image layer in the SVG file. Thus, the file retains a permanent record of the contrasts used to define each ROI.

## 5. Future development

The pycortex WebGL view provides an unprecedented method for exploration of cortical MRI data. The interactive interface allows results to be manipulated in innovative ways that facilitate comprehension, and the ability to generate static views greatly simplifies data sharing and publication. However, the current WebGL viewer contains a limited set of plugins for interactive data visualization. We plan to develop a large set of interactive plotting tools that will facilitate dynamic data analysis in a web browser. We are also working on extensions to pycortex that allow ECoG and EEG data to be visualized on the cortical surface. Unfortunately because WebGL is a very new standard, support is still unreliable. Therefore, we are also exploring options to stabilize the software on additional platforms (including mobile platforms) and to improve accessibility.

## Author contributions

JSG designed the software; JSG, AH, and ML contributed code; JSG, AH, ML, and JLG wrote the paper.

## Funding

This work was supported by the National Eye Institute (EY019684 and EY022454), NIH NEI F32EY021710 to ML, and from the Center for Science of Information (CSoI), an NSF Science and Technology Center (IIS-1208203), under grant agreement CCF-0939370.

## Conflict of interest statement

The authors declare that the research was conducted in the absence of any commercial or financial relationships that could be construed as a potential conflict of interest.

## Footnotes

^1^https://surfer.nmr.mgh.harvard.edu/fswiki/FreeSurferWiki?action=AttachFile&do=get&target=FreeSurfer_Suggested_Morphometry_Protocols.pdf
